# A hierarchical approach for speech-instrumental-song classification

**DOI:** 10.1186/2193-1801-2-526

**Published:** 2013-10-17

**Authors:** Arijit Ghosal, Rudrasis Chakraborty, Bibhas Chandra Dhara, Sanjoy Kumar Saha

**Affiliations:** CSE Dept., Institute of Technology and Marine Engg, Diamond Harbour, West Bengal India; Indian Statistical Institute, Kolkata, India; Department of Information Technology, Jadavpur University, Kolkata, India; Department of Computer Science & Engineering, Jadavpur University, Kolkata, India

**Keywords:** Speech/Music classification, Instrumental/Song classification, Audio texture, Mel frequency cepstral co-efficient, Random sample and consensus

## Abstract

Audio classification acts as the fundamental step for lots of applications like content based audio retrieval and audio indexing. In this work, we have presented a novel scheme for classifying audio signal into three categories namely, speech, music without voice (instrumental) and music with voice (song). A hierarchical approach has been adopted to classify the signals. At the first stage, signals are categorized as speech and music using audio texture derived from simple features like ZCR and STE. Proposed audio texture captures contextual information and summarizes the frame level features. At the second stage, music is further classified as instrumental/song based on Mel frequency cepstral co-efficient (MFCC). A classifier based on Random Sample and Consensus (RANSAC), capable of handling wide variety of data has been utilized. Experimental result indicates the effectiveness of the proposed scheme.

**Electronic supplementary material:**

The online version of this article (doi:10.1186/2193-1801-2-526) contains supplementary material, which is available to authorized users.

## 1 Introduction

With the rapid growth in multimedia technology, it has become quite easy to possess an audio library of huge volume. But, the management of such database becomes very crucial for efficient use. The collection, classified into different categories like speech, music may render better organization and will provide an easy access to desired data. But, the manual classification is quite labor intensive. As a result, automatic classification and retrieval of audio data has become an active area of research. A lot of work have been directed towards the development of content-based image and video retrieval system in comparison to audio domain (Zhang and Kuo 1998). An efficient audio classification system can serve as the foundation for various applications like audio indexing, content based audio retrieval (Tseng 1999), music genre classification (Tzanetakis and Cook 2002; Lidy and Rauber 2005), audio content description.

In general, an automatic audio classification system consists of two steps: *extraction of features from the signal* and *classification based on the extracted feature*. In order to discriminate speech and music, a wide variety of *low level* and *perceptual/psycho-acoustic features* have been proposed by the researchers to describe the audio content. Low level features can further be categorized as *time domain* and *frequency domain* features. ZCR (zero crossing rate) (West and Cox 2004; Downie 2004) and STE (short time energy) (Saunders 1996; El-Maleh et al. 2000) are the commonly used time domain features. Features like signal bandwidth, spectral centroid, signal energy (Beigi et al. 1999; McKay and Fujinaga 2004; West and Cox 2005), fundamental frequency (Zhang and Kuo 1998), mel-frequency cepstral co-efficients (MFCC) (Eronen and Klapuri 2000; Foote 1997) belong to the category of frequency domain features. Roughness and loudness measures (Fastl and Zwicker 2007) have been presented to capture the perceptual aspect. In (Breebaart and McKinney 2004), a model has been proposed to simulate the human auditory system and 62-dimensional features are computed to describe the auditory filter-bank temporal envelope (AFTE). Sub-band energy (Liu et al. 1997; Guo and Li 2003) is used as descriptor. Compressed domain features (Wang et al. 2003) have also been introduced.

Different classification techniques have been adopted to categorize audio data into various classes. Threshold based techniques are widely used (Saunders 1996; Zhang and Kuo 2001). Audio classification based on neural network (Matityaho and Furst 1994; Harb and Chen 2003), genetic algorithm (Pwint and Sattar 2005), SVM (Guo and Li 2003; Sadjadi et al. 2007), Hidden Markov Model (Pikrakis and Giannakopoulos 2006) have also been considered by the researchers.

It has been observed that lot of attempts have been made to classify the audio data in a wide variety of categories and sub-categories. But, in most of the cases sub-classification of music data into song (music with voice) and instrumental (music without voice) has been ignored. Only a few works (Berenzweig and Ellis 2001; Zhang and Kuo 2001; Zhang 2003) have addressed the issue. Berenzweig et al. (2001) have relied on the fact that song will have features of speech embedded in it and a speech-trained model is used to detect the song. But, the success of the scheme depends on the availability of suitable speech recognizer. Zhang et al. (2001) have considered four features based on average ZCR and fundamental frequency. Threshold for each features are considered to characterize the music signals and finally the decision is taken based on heuristic approach. A modified version of the scheme is presented in (Zhang 2003). The success of the schemes heavily depend on the proper selection of thresholds.

Past study indicates that researchers have experimented with a wide variety of features and classification techniques. In our effort, we have relied on the basic perception of the audio signal of each type and accordingly features are considered to represent their acoustic signatures. We have proposed audio texture as a means of concise representation of the audio signal. Normally, features are computed over the frames in the signal. Audio texture captures the repetitive pattern of such features. In this work, ZCR and STE have been considered as the frame based features. We have also included the task of differentiating music without voice (instrumental) and with voice (song) in our system. It is important in the applications like locating singing voice segments in music signal or in the audio track of a movie (Berenzweig and Ellis 2001; Zhang and Kuo 2001). Moreover, in the context of a multilingual nation like India, it is often required for a music retrieval system to discriminate between music with voice and music without voice. Thus, the proposed system classifies the signals into three classes namely, *speech*, *instrumental* and *song*. The paper is organized as follows. The introduction is followed by the proposed methodology described in Section 2. Experimental result and concluding remarks are put in Section 3 and 4 respectively.

## 2 Proposed methodology

As it has been discussed in Section 1, two class problem of speech and music discrimination has been addressed by the researchers based on different low level features. But, a direct application of those schemes can not handle the problem of three way classification *i.e.* identification of speech, instrumental and song. The difficulty arises out of the fact that in the feature space, song has a substantial overlap with speech and instrumental as it is composed of both the components. This observation has motivated us to go for hierarchical approach. At the first stage we opt for classifying the signal into speech and music and in the subsequent stage we take up the issue of categorizing music into instrumental and song. We rely on audio texture based on ZCR and STE (Ghosal et al. 2009) and MFCC based features respectively in the two stages. Proposed audio texture provides an effective mechanism for summarizing the ZCR and STE values of all the frames in the audio signal. The computation of the features have been elaborated in Section 2.1 and 2.2. The classification scheme is described in Section 2.3.

### 2.1 Audio texture

The concept of texture in the domain of image processing is quite common. For an image, texture is formed by the repetition of fundamental image elements and it is evaluated by the properties like coarseness, smoothness, randomness and regularity. In an intensity image, intensity variation over a neighbourhood gives rise to the texture and co-occurrence of gray levels has evolved as a measure (Haralick and Shapiro 1991). The idea has been further extended in (Saha et al. 2004) where instead of dealing with the pixel intensity, co-occurrence of features at sub-image level has been considered to get a better perception. We have adopted the similar concept and proposed audio texture for characterizing an audio signal.

In general a speech signal occupies a limited range of frequencies in comparison to a music signal. A speech signal is typically characterized by the presence of voiced and unvoiced zone and they differ in terms of frequency and energy. Moreover, silence is quite common in a speech signal and such zones are of almost zero energy. Thus, in case of a speech signal, interleaved occurrences of voiced, unvoiced and silence zone gives rise to a pattern. In case of music, such behaviour is absent. This observation has led us to devise audio texture for speech/music classification. The zones are taken as the the fundamental elements of the speech signal and repetition of those elements leads to audio texture. As the zones are distinguished by the features like frequency and energy content, we consider ZCR and STE computed over the frames as an approximation of the zone features. The repetition pattern captured in the co-occurrence matrices of such features can act as a measure for audio texture.

In Section 1, it has been indicated that zero crossing rate (ZCR) and short time energy (STE) are two commonly used time domain, low level features which play major role in speech/music discrimination. Texture of the audio signal is generated based on those. Considering audio data as discrete signals, it is said that a zero crossing has occurred whenever two successive samples have different signs. Rate of zero crossing provides an impression regarding the frequency content. Audio signal is divided into *N* frames { *x*_*i*_(*m*): 1 ≤ *i* ≤ *N*}. Then, for *i*^*th*^ frame, zero crossing rate is computed as follows:

1 $$z_{i}=\overset{n-1}{\underset{m=1}{\sum }}\text{sign}[\;x_{i}(m-1)x_{i}(m)]$$

n is the number of samples in the *i*^*th*^ frame and

2 $$\text{sign}[\;v]=\left\{\begin{array}{ll} 1, & \text{if}\;v<0\\ 0, & \text{otherwise} \end{array}\right.$$

As the collection of frame level ZCR is of very high dimension, the audio signal is represented by the summarized information. Mean and standard deviation of { *z*_*i*_:*i* = 1,2,…, *N*} are taken as two features. Such type of representation gives only an overall idea about the signal. To obtain a better representation of the signal characteristics we have utilized the concept of co-occurrence matrix (Haralick and Shapiro 1991) which is widely used in image processing. In an image, the occurrence of the different intensity values within a neighborhood reflects a pattern and it is utilized to parameterize the appearance/texture of an image. The same concept is adopted here. For each frame, ZCR is computed using equation (1). Thus, { *z*_*i*_}, a sequence of ZCR is obtained for the signal. Occurrence of different ZCR values within a neighborhood reflects the pattern and characterizes the quasi-periodic behavior of the signal. Thus, a matrix, *C* of *L* × *L* dimension (where, *L* = *max*{*z*_*i*_} + 1) is formed as follows:

-  Initialize *C*[ *i*][*j*] = 0 ∀ *i*, *j* ∈ {0,1,…, *L*}
-  for i = 1 to *N* - *dC*[*z*_*i*_][*z*_*i* + *d*_] = *C*[*z*_*i*_][*z*_*i* + *d*_] + 1
-  $C[\;i][j]=\frac{C[i][j]}{\underset{r}{\sum }\underset{c}{\sum }C[r][c]}\forall i,j\in \{0,1,\ldots ,L\}$

where, *d* is the distance at which occurrence of the values are being considered. Thus, the matrix *C* represents distribution of pairwise occurrence of different ZCR values. It is likely that in case of a speech signal, there will be substantial co-occurrence of low ZCR denoting silence zone and high-low transition (or vice versa) for non-silence to silence (or vice versa) switching. Such transition also occurs due to interleaving of voiced and unvoiced speech. These will have a reflection in *C*. Music is comparatively richer in frequency content, distribution will be well spread in the matrix. Due to noise there may be little variation in the signal which may affect the co-occurrence matrix. Moreover, very close frequencies are also not perceivable to human ear.

To combat these issues, we had to go for a modified scheme to construct the co-occurrence matrix. The ZCR scale may be divided into *k* bins defined by the points *μ*_*z*_ ±*t* × *s* × *σ*_*z*_ where, *μ*_*z*_ and *σ*_*z*_ are mean and standard deviation of { *z*_*i*_}, *t* takes the values 0,1,2,… and *s* is the step size. It is obvious that substantial contribution will be confined within *μ*_*z*_ ± *σ*_*z*_. Hence, to reveal the distribution characteristics in a detailed manner *s* is taken from (0, 1). Once the bins have been formed, *z*_*i*_s are mapped onto bins and instead of *z*_*i*_ values, corresponding bin numbers are used as the index in forming the co-occurrence matrix *M*_*k* × *k*_. From the co-occurrence matrix, *M*, following statistical features (Umbaugh 2005) are computed:

3 $$\begin{array}{ll} \text{Entropy} & =-\underset{i}{\sum }\underset{j}{\sum }M[\;i][j]{\text{log}}_{2}M[\;i][j]\; \end{array}$$

4 $$\begin{array}{ll} \text{Energy} & =\underset{i}{\sum }\underset{j}{\sum }{\left[\;M[\;i][j]\right]}^{2}\; \end{array}$$

5 $$\begin{array}{ll} \text{Inertia} & =\underset{i}{\sum }\underset{j}{\sum }{\left(i-j\right)}^{2}M[\;i][j]\; \end{array}$$

6 $$\begin{array}{ll} \text{Inverse}\_\text{difference} & =\underset{i}{\sum }\underset{j}{\sum }\frac{M[\;i][j]}{\left|i-j\right|},i\neq j\; \end{array}$$

7 $$\begin{array}{ll} \text{Correlation} & =\;\frac{1}{\sigma_{x}\sigma_{y}}\;\underset{i}{\sum }\;\underset{j}{\sum }(i\;-\;\mu_{x})(j\;-\;\mu_{y})M[\;i]\;[j]\; \end{array}$$

where,

$$\begin{array}{l} \mu_{x}=\underset{i}{\sum }i\underset{j}{\sum }M[\;i][j]\\ \mu_{y}=\underset{j}{\sum }j\underset{i}{\sum }M[\;i][j]\\ \sigma_{x}^{2}=\underset{i}{\sum }{\left(1-\mu_{x}\right)}^{2}\underset{j}{\sum }M[\;i][j]\\ \sigma_{y}^{2}=\underset{j}{\sum }{\left(1-\mu_{y}\right)}^{2}\underset{i}{\sum }M[\;i][j] \end{array}$$

Thus, computing these features, a 5-dimensional ZCR based feature vector is formed. It may be noted that the texture features thus obtained is a better alternative for summarizing the frame level features.

Similarly, short time energy based features are also computed. First of all, for each frame short time energy is computed as follows:

8 $$E_{i}=\frac{1}{n}\overset{n-1}{\underset{m=0}{\sum }}{\left[\;x_{i}(m)\right]}^{2}$$

where frame contains *n* samples. Based on the set of STE, *E*_*i*_ for the frames, the co-occurrence matrix is formed in the same manner as it has been done in case of co-occurrence matrix of ZCRs. As the range of energy values is quite high, it would have been a big problem for matrix dimension. Mapping of the absolute value to bin solves the problem. Such mapping also overcome another problem. Overall rise/fall in the amplitude level of the signal does not change the nature of the signal but affects the energy value. Mapping scheme present in this work also cancels such impact and retains the signal characteristics.

**Figure 1:**
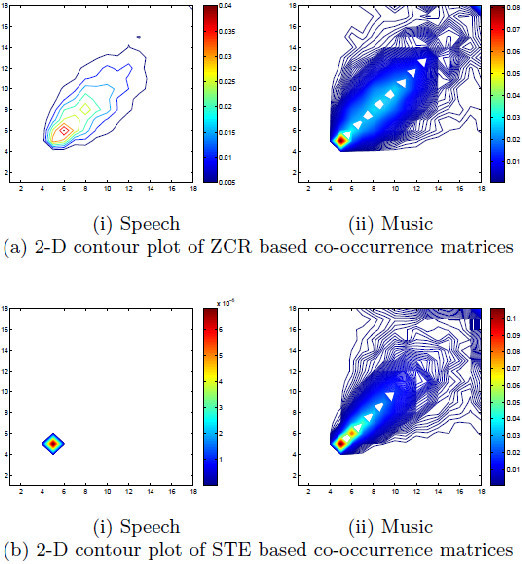
**2-D contour plot of co-occurrence matrices of speech and music signals – (a) ZCR based co-occurrence matrices (b) STE based co-occuurence matrices.** X and Y axes show ZCR(STE) bins and values of the matrix elements are denoted by different colours.

In case of a speech signal, silence zone will have minimal energy. Moreover, interleaved voiced and unvoiced speech will lead to interleaving of high and low energy. It will give a typical pattern in the co-occurrence matrix enabling us to discriminate the speech from rest. Co-occurrence matrix based features are computed to obtain 5-dimensional STE based feature vector. Figure 1 show 2-D contour plots of ZCR co-occurrence matrices and STE co-occurrence matrices. Frequency of the co-occurrence pattern has been indicated by the colour and colour code also has been shown along with. It is clear that the plots are quite different for speech and music signals. For ZCR occurrence patterns of speech signal shows a few peaks as its frequency content is limited. Music being rich in frequency content, multiple peaks becomes apparent in the form of coloured patch in the plot. For speech silence zone has almost zero energy and energy distribution is localized with in a small range of bins. In case of music, energy is distributed widely across the bins which is reflected by the coloured patch in the plot. Thus, the utility of the concept of occurrence pattern is clearly visible.

Taking ZCR and STE co-occurrence matrices based features together, a 10-dimensional feature vector is formed and it acts the descriptor for an audio signal for speech/music classification. As the proposed audio texture for music with/without voice is quite similar in nature, it can not be used for discriminating them further and it has forced us to restrict its usage in speech/music classification only.

### 2.2 MFCC

It has been indicated in (Zhang and Kuo 2001) that unlike song, the spectrogram of instrumental music reflects stable frequency peaks in the spectrogram. In case of song, because of the human voice, such stability is not visible. It has motivated them to think of ZCR and fundamental frequency based descriptors and to devise a threshold based classification scheme. The same observation has motivated us to look into frequency domain. In case of instrumental music, ideally the spectral power is confined around certain frequencies. Whereas, for song, it is distributed over a wider range of frequency. Song is further complex signal as it is normally accompanied by instrumental music also. Considering all these aspects, we have relied on cepstrum based feature. A cepstrum is the inverse Fourier transform of the log spectrum and the technique is particularly good at separating the components of complex signals made up of several simultaneous but different elements combined together.

Mel-frequency cepstral co-efficients are short term spectral based features used by many researchers for speech recognition (Walker et al. 2004), retrieval system (Foote 1997), music summarization (Logan and Chu 2000), speech/music discrimination (Logan 2000). The strength of MFCC is in compact representation of amplitude spectrum. The steps for computing MFCC is elaborated in (Rabiner and Juang 1993). The brief description is as follows.

The audio signal is first divided into number of frames of fixed duration. Frames may consist of samples with an overlap with the previous frame. To minimize the discontinuity at the beginning and end of the frame an windowing function (Hamming window is the most widely used one) is also applied on the frame. Amplitude spectrum for each (windowed) frame is obtained by applying Discrete Fourier Transform (DFT). As the relation between perceived loudness and amplitude spectrum is more logarithmic than linear, logarithm of amplitudes is taken. Thus, *N*- dimensional spectrum is obtained where *N* is the frame size. The spectrum is smoothened to make it perceptually meaningful. The simplest way of doing this to consider the average spectrum over the frequency bins. But eqi-spaced bin over the frequency scale does not conform the human auditory system as the perceived frequency and the signal frequency are not linearly related. It has led to the development of Mel frequency. The relation can be expressed as follows.

$$f_{m}=2595*{\text{log}}_{10}(1+\frac{f}{100})$$

where, *f* and *f*_*m*_ are signal frequency and corresponding Mel frequency respectively. The mapping is approximately linear below 1kHz and logarithmic above. Thus, logarithm of amplitude spectrum obtained after DFT is mapped on to Mel-frequency scale and smoothened by considering the bins over the Mel-scale. The elements in the smoothened Mel-spectra vector are highly correlated. To decorrelate and to reduce the number of parameters Discrete Cosine Transform (DCT) is performed as follows.

$$k[\;n]=\sqrt{\frac{2}{N_{T}}}C(n)\overset{N_{T}-1}{\underset{k=0}{\sum }}S[\;k]\text{cos}\frac{\pi (2k+1)n}{2N_{T}}$$

where

$$C(n)=\left\{\begin{array}{ll} \frac{1}{\sqrt{2}}, & \text{if}\;n=0\\ 0, & \text{otherwise} \end{array}\right.$$

where, 0 < *n* < *N*_*T*_ - 1, *S*[ *k*] be the Smoothened Mel-spectrum and *N*_*T*_ is the number of elements in the smoothened Mel-spectra vector. *k*[ *n*], thus obtained denotes the Mel-frequency cepstral co-efficient and first 13 co-efficients are taken as the features for the frame.

After computing the MFCCs for all the frames, the vector comprising of the average value corresponding to each co-efficient forms the feature descriptor. It may be noted that each Mel-frequency cepstral co-efficient, *k*[ *n*] is obtained after DCT of log-spectrum and hence, it captures the weighted combination of all spectral component. As a result, even if a limited number of co-efficients are taken as features, signature of the complete frequency spectrum is still embedded in them. Thus, MFCC provides a compact representation of the amplitude spectrum of a signal.

**Figure 2:**
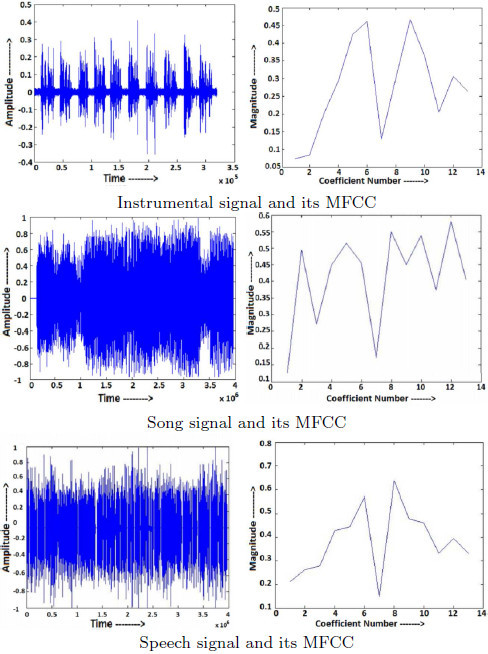
**Different types of audio signals and their MFCC plots.**

It has been already mentioned that song is complex in nature in comparison to instrumental and unlike song, instrumental is characterized by stable frequency peaks in the spectrogram (Zhang and Kuo 2001). The presence of perceivable amplitude over a wider range of frequency in a song is well reflected by the presence of more number of strong peaks in the MFCC plots as shown in Figure 2. As the plots for instrumental and song is quite different, MFCC can be utilized in classifying the music signals in the sub categories in the second stage of the proposed scheme. On the other hand, the MFCC plots for speech signals, there are few stable peaks making it similar to instrumental signal and also a number of weak peaks which may overlap with song signal. Thus, it may create confusion with instrumental/song signal in various cases. As a result its success in discriminating speech from rest is limited and we have avoided using it at first stage. Moreover, it can not help in a direct classification of speech, instrumental and song.

### 2.3 Classification scheme

The audio signal, be it speech or instrumental or song, may have a wide variety making the task of classification critical. In this context a direct threshold based approach is quite prohibitive. As a result, many researchers have relied on the classification schemes like neural network, SVM and HMM. The variation even within a class of audio signal gives rise to outliers putting detrimental bias in classification. Furthermore, each classifier has its own set of parameters which are not always readily perceivable and performance depends heavily on the proper selection of such parameters. In order to achieve optimal performance, tuning of parameters like kernel width is very critical for SVM. On the other hand, in case of HMM, finding out number of states, state transition probability, distinct observation per state etc. are not at all trivial task. It has motivated us to look for an estimator which is characterized by the the parameters those are easy to interpret and tune and capable of handling the diversity of data satisfactorily. In this context, RANdom Sample And Consensus (RANSAC) appears as a suitable alternative that can model the diversified data even in presence of considerable outliers. Successful application of the scheme in the domain of image processing (Torr and Zisserman 2000; Zuliani et al. 2005) has also motivated us to apply it for audio classification.

RANSAC (Fischler and Bolles 1981) is an iterative method to estimate the parameters of a certain model from a set of data contaminated by large number of outliers. The strength of RANSAC over other estimators lies in the fact that the estimation is made based on inliers *i.e.* whose distribution can be explained by a set of model parameters. It can produce reasonably good model provided a data set contains a sizable amount of inliers. It may be noted that RANSAC can work satisfactorily even with outliers amounting to 50% of entire data set (Zuliani 2005).

RANSAC algorithm is primarily composed of two steps – *hypothesize* and *test*. These are executed iteratively. During hypothesize phase, a minimal sample set is randomly selected and model parameters are computed based on only the elements in the selected sample set. In the test phase, consistency of all the elements in the entire data set are verified to check whether or not they are consistent with the model obtained. Consistent elements are included to form the new sample set. The process goes on iteratively and in each iteration a model is obtained. Finally, the model that fits best is taken as the estimate.

Considering the data elements to be *n*-dimensional, RANSAC tries to fit a hyperplane and estimates the model parameters. Let the hyperplane is represented as

$$w_{0}+w_{1}d_{1}+w_{2}d_{2}+\ldots +w_{n}d_{n}=0$$

where, < *d*_1_, *d*_2_,… *d*_*n*_ > be the *n*-dimensional point. It estimates the value of *w*_*i*_s by minimizing the fitting error for each element in the entire data set. An element *e*_*i*_ is considered as an inlier/consistent with respect to the model provided its orthogonal regression, *d*(*e*_*i*_, *W*) with the model is within the threshold *δ*; where,

$$d(e_{i},W)=\frac{w_{0}+\sum_{j}w_{j}e_{\text{ij}}}{\sqrt{w_{0}^{2}+\sum_{j}w_{j}^{2}}}$$

and *e*_*i*_ = < *e*_*i* 1_, *e*_*i* 2_ … *e*_*in*_ >. In our experiment, *δ* is taken as 0.02 as suggested in (Fischler and Bolles 1981). $\sum d(e_{i},W)$ is taken as the total fitting error for the model under consideration. The model with minimum error is considered as the final one.

Classically, RANSAC is an estimator for the parameters of a model from a given data set. But, in this work, it has been used a classifier. Corresponding to the data set of each category a model is estimated first. Then for a given element, its class can be determined easily by finding the best matched model. As it has been discussed that RANSAC estimates the model relying on the inliers, unlike other technique, it is less affected by the noisy data. Thus, RANSAC is well suited for our purpose.

It has been already discussed that audio texture can discriminate only speech and music. It can not further distinguish a music signal as instrumental/song. On the other hand MFCC plots for instrumental and song are quite distinct but the same for speech introduces confusion. Hence at first stage RANSAC models the signals as speech or music considering audio texture as the feature and classification is carried out. At the next stage, RANSAC further classifies the detected music signals into song/instrumental based on the model formed using MFCC as the feature. Thus, a hierarchical classification scheme is followed.

## 3 Experimental result

In order to carry out the experiment, we have prepared a database consisting of 399 speech files and 540 music (270 instrumental and 270 song) files. Files are mostly CD recordings, some are the recordings of live program and noise affected. Part of data has also has been downloaded from different web sites. Speech data differs in terms of speaker and language. Instrumental files correspond to different instruments like piano, guitar, flute, drum. The songs are of different genre like classical, folk, rock. The database thus reflects appreciable variety in each class. Each file has the audio of around 40-45 seconds duration. Sampling frequency for the data is 22050 Hz. Samples are of 16-bits and of type mono.

In our experiment, apart from RANSAC, we have considered other classification techniques like K-means clustering, multilayer perceptron network (MLP), support vector machine (SVM). For all the classifiers, we need training data and test data. We have carried out two set of experiments. In one case (referred as T1), 50% of the data for each class has been used as training data and rest are taken as test data. In the other case (referred as T2), 25% of the data for each class is taken as training data and rest form the test data.

In case of RANSAC, training data used for generating the model and remaining data are used for testing. In T1, experiment is repeated by reversing the training and test data set. Finally, average of the performances is considered. For T2, four sets of training data is considered and experiments are carried out four times by considering the remaining data in each case as the test data. Finally, as in case of T1, average of the performances is reported. Similar approach is followed for K-means clustering, MLP and SVM based classification.

For K-means clustering based classification (Hastie et al. 2005), training data is first clustered with number of clusters same as the number of categories into which classification is required. Clusters are labeled based on the type of the majority data elements in the cluster. Test data is classified following nearest neighbour classification rule. For MLP, we have considered only one hidden layer. Number of nodes in the input and output layers are *n*_1_ and *n*_2_ respectively. *n*_1_ is taken as the dimension of the feature vector and *n*_2_ is same as the number of classification labels. Hidden layer consists of $\frac{n_{1}+n_{2}}{2}+1$. In order to train the network, feed forward back propagation method has been used. For SVM, we have considered Radial Basis Function (RBF) as the kernel function. Cost parameter controlling the number of support vectors and gamma that governs the kernel width are two important parameters to be tuned. In our experiment, the optimal values for the parameters are chosen by applying grid search over a wide range of possible values.

Initially, we have tried to classify the audio signals directly into three categories namely, speech, instrumental and song. Table 1 shows the overall classification accuracy. Experiments have been carried out using only 10-dimensional audio texture (referred as A) and again using 13-dimensional MFCC based features (referred as B). Finally, both type of features are taken together to form 23-dimensional feature vector (referred as A+B). ZCR and STE computed over each frame are divided by the frame size to normalize the values. In case of MFCC, at frame level the co-efficients are first computed and average of the corresponding co-efficients over the frames are taken as the signal level descriptor. 13-dimensional feature vector is then normalized where the strongest magnitude is mapped to 1 and others are mapped proportionately. Thus, each element in the feature vector is normalized with in the range [ 0,1]. It has been discussed in Section 2 that proposed audio texture can discriminate speech from the rest and MFCC based features are similar for speech and music. As a result, a direct three way classification by audio texture, MFCC or their combination does not perform well irrespective of the classification technique as indicated in Table 1. It has motivated us to go for hierarchical approach.

**Table 1:** **Overall accuracy (in %) for direct classification into speech, instrumental and song**

	T1			T2		
Classification	Audio	MFCC	A+B	Audio	MFCC	A+B
scheme	texture	(B)		texture	(B)	
	(A)			(A)		
K-means	54.00	52.67	54.88	51.19	50.00	52.67
MLP	62.00	71.78	60.00	60.68	69.14	59.20
RANSAC	79.77	72.22	79.33	78.88	70.18	79.08

As it has been discussed in Section 2.1, proposed audio texture based on ZCR and STE can clearly discriminate speech and music signal, we have taken up the issue of speech-music classification at the first stage. Performance of proposed feature has been compared with the commonly used ZCR and STE based features (the average and standard deviation of the frame level ZCR and STE values). Table 2 shows that proposed features perform better for different classification schemes. Thus, it is effective in summarizing the frame level features. We have also worked with the concept of delta and double delta (*Δ* - *Δ*^2^) of features (Kumar et al. 2011; Chen and Bilmes 2007) as it takes care of contextual information like the proposed feature. Similar process is repeated with frame level delta co-efficients to generate double delta features. Thus, 26 dimensional feature vector is obtained. Performance of this feature as shown in Table 2 reflects that it works better in recognizing speech in comparison to music recognition. But, the result indicates that proposed feature is more effective. It may be noted that if the volume of training data is reduced from 50% (in case of T1) to 25% (in case of T2), there is a fall in accuracy to an extent varying between 2% to 5%. Even then, the proposed feature provides better result. It is also evident that RANSAC based classification provides quite satisfactory performance.

**Table 2:** **Accuracy (in %) of speech, music classification**

Classification scheme	Experiment setup	Type of signals		Featutre set	
			ZCR, STE based features	***Δ***- ***Δ***^2^	Proposed audio texture
K-means	T1	Speech	50.55	60.00	73.89
		Music	74.07	74.07	85.93
		Overall	64.67	68.08	81.11
	T2	Speech	48.15	57.52	71.85
		Music	71.04	72.10	84.40
		Overall	61.87	65.91	79.38
MLP	T1	Speech	71.11	78.50	78.33
		Music	90.37	75.92	88.15
		Overall	82.67	77.02	84.22
	T2	Speech	68.52	74.92	74.07
		Music	86.63	72.84	84.40
		Overall	79.38	73.72	80.27
SVM	T1	Speech	73.89	86.00	78.33
		Music	90.74	81.48	89.26
		Overall	84.00	83.40	84.89
	T2	Speech	69.63	83.28	76.67
		Music	88.86	79.75	85.40
		Overall	81.16	81.25	81.90
RANSAC	T1	Speech	75.00	88.00	96.11
		Music	92.96	85.93	97.78
		Overall	85.78	86.80	97.11
	T2	Speech	74.07	85.62	93.70
		Music	90.59	82.47	93.81
		Overall	83.98	83.81	93.77

We have further compared the performance of the proposed system with the work done by Sadjadi et al (2007). In their work, 16-dimensional feature vector using FFT based perceptual features and MFCC has been considered and one class SVM based iterative method for clustering has been deployed. Table 3 shows that proposed methodology performs better under both the training condition.

**Table 3:** **Comparison of performance for speech, music classification**

	Classification accuracy (in %)					
Methodology	Speech		Music		Overall	
	T1	T2	T1	T2	T1	T2
Sadjadi’s method	88.89	86.67	84.44	82.67	86.22	84.27
(Sadjadi et al.2007)						
Proposed method	95.55	93.70	97.78	93.81	96.89	93.77

With the data set classified as music, we carry out the second stage of classification. At this stage, music signal is further classified as music without voice (instrumental) and music with voice (song). As discussed in Section 2.2, 13-dimensional MFCC based feature vector is used. Table 4 shows the classification accuracy for various schemes and training conditions. It has been observed that accuracy falls to an extent for smaller training data set. Even then, the proposed scheme provides better and considerably high accuracy.

**Table 4:** **Accuracy (in %) of instrumental, song classification**

Classific. scheme	Instrumental		Song		Overall	
	T1	T2	T1	T2	T1	T2
K-means	40.07	39.60	92.60	87.13	66.67	63.37
MLP	62.22	59.41	94.07	89.60	78.15	74.50
SVM	77.04	74.75	82.96	81.68	80.00	78.21
RANSAC	94.81	90.59	91.11	88.61	92.96	89.60

It has been indicated in Section 1 a very few work have been directed in discriminating instrumental and song. Zhang (2003) in his work has addressed the issue. The classification has been made based on the average zero crossing rate and fundamental frequency properties. Four aspects like degree of a signal being harmonic, fundamental frequency’s concentration during a period of time, variance of ZCR and amplitude range of average ZCRs have been considered. For each of these four aspects, there is one empirical threshold set and a decision value defined. If the threshold is satisfied, the decision value is set to 1; otherwise, it is set to a value between 0 and 1 according to the distance to the threshold. The four decision values are averaged with predetermined weights to derive a total probability for a music to be instrumental. It is taken as instrumental if the said probability exceeds certain threshold and at least three of the decision values are above 0.5. The method heavily relies on the selection of number of parameters like thresholds and weights. It is quite difficult to set them properly. Moreover, details regarding the threshold have not been outlined. Hence, In order to compare the performance, we have considered the features proposed by Zhang and tried with different classification techniques. Table 5 indicates, proposed methodology performs better.

**Table 5:** **Comparison of performance (in %) for instrumental, song classification**

Methodology	Instrumental		Song		Overall	
	T1	T2	T1	T2	T1	T2
Zhang’s feature						
and SVM	64.44	62.87	82.22	80.69	73.33	71.78
(Zhang 2003)						
Zhang’s feature						
and RANSAC	74.81	73.27	77.78	76.73	76.30	75.00
(Zhang 2003)						
Proposed method	94.81	90.59	91.11	88.61	92.96	89.60

## 4 Conclusion

In this work, we have presented a hierarchical scheme for classifying audio signals into three categories namely speech, music without voice (instrumental), music with voice (song). In the first stage, we classify the signals as speech or music and subsequently music is further classified as instrumental and song. Audio texture that has been derived based on ZCR and STE co-occurrence matrices can successfully discriminate speech and music. Thus, audio texture acts as an effective way for summarizing frame level features. It has been shown that MFCC based features are well suited in classifying the music signal as instrumental and song. RANSAC has been utilized as the classifier and it is capable of handling the wide variation in data. Experimental result indicates the effectiveness of the proposed methodology.

## Authors’ original submitted files for images

Below are the links to the authors’ original submitted files for images. Authors’ original file for figure 1 Authors’ original file for figure 2 Authors’ original file for figure 3 Authors’ original file for figure 4 Authors’ original file for figure 5 Authors’ original file for figure 6 Authors’ original file for figure 7 Authors’ original file for figure 8 Authors’ original file for figure 9 Authors’ original file for figure 10
