# Supplementary figures and images for: A hierarchical approach for speech-instrumental-song classification
Source: Springerplus. 2013 Oct 17;2(1):526. doi: 10.1186/2193-1801-2-526 (PMC4322669; doi:10.1186/2193-1801-2-526)

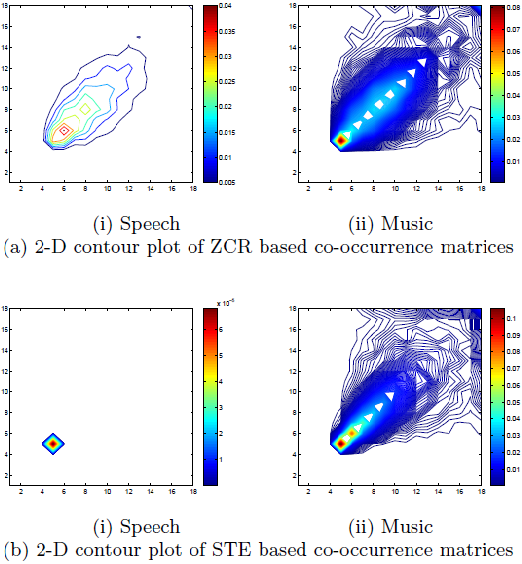

Supplement: Supplementary file 1 — Authors’ original file for figure 1 [file 40064_2013_1422_MOESM1_ESM.tif]

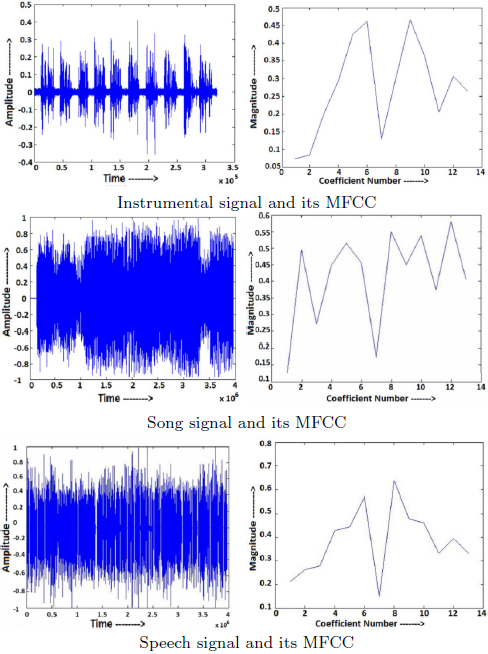

Supplement: Supplementary file 2 — Authors’ original file for figure 2 [file 40064_2013_1422_MOESM2_ESM.tif]
